# Supplementary material for: Fungal mycelium classified in different material families based on glycerol treatment
Source: Commun Biol. 2020 Jun 26;3:334. doi: 10.1038/s42003-020-1064-4 (PMC7320155; doi:10.1038/s42003-020-1064-4)
Supplement: Supplementary file 1 — Supplementary Information [file 42003_2020_1064_MOESM1_ESM.pdf]

Supplementary Information

Fungal mycelium classified in different material families based on glycerol treatment

Freek VW Appels, Jeroen G van den Brandhof, Jan Dijksterhuis, Gijs W de Kort, Han AB Wösten

Supplementary Figures

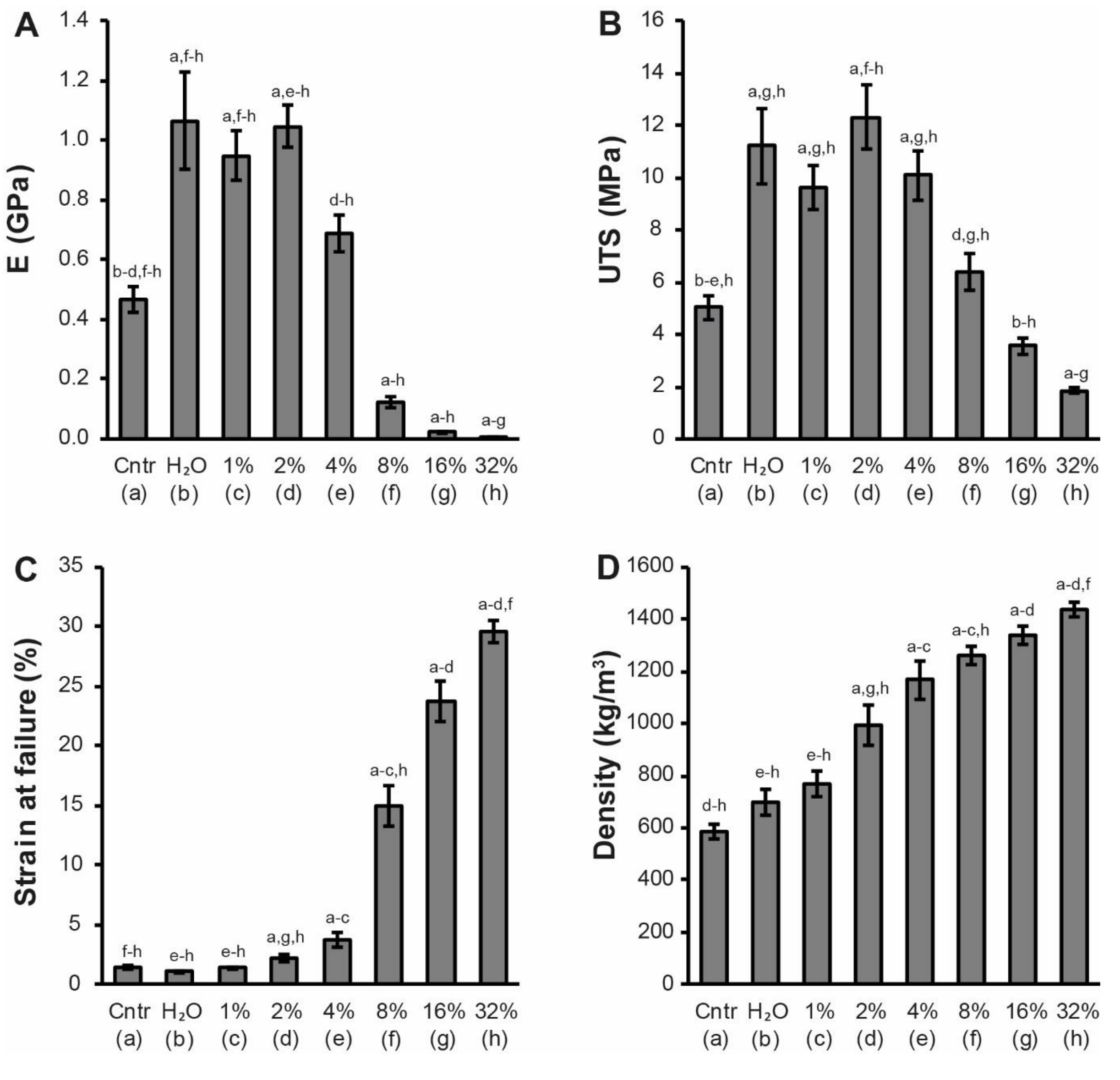

Supplementary Figure 1. Material properties of mycelial films of *S. commune*. Young's modulus (E) (**A**), ultimate tensile strength (σ) (**B**), strain at failure (ε) (**C**) and density (**D**) of *S. commune* mycelium films treated with 0–32% glycerol, untreated mycelium films serving as a control. Letters above bars indicate statistically significant differences to the materials specified with that letter as was determined by a Welch's *t*-test followed by a Games-Howell post hoc test ($p \leq 0.05$). The number of biological replicates per treatment is shown in Table 1. Bars represent mean ± SEM.

Supplementary Tables

Supplementary Table 1. Thickness increase resulting from water submersion of mycelial films of *S. commune.* Thickness increase of mycelium films of *S. commune* treated with water or different concentrations of glycerol after 24 hours water submersion (mean ± SEM, *n*=4).

	Thickness increase
Control	102±8%
H_2O	493±33%
1% glyc	496±16%
2% glyc	472±55%
4% glyc	450±21%
8% glyc	331±17%
16% glyc	277±18%
32% glyc	189±19%
