# Supplementary material for: Fungal mycelium classified in different material families based on glycerol treatment
Source: Commun Biol. 2020 Jun 26;3:334. doi: 10.1038/s42003-020-1064-4 (PMC7320155; doi:10.1038/s42003-020-1064-4)
Supplement: Supplementary file 3 — Description of Additional Supplementary Files [file 42003_2020_1064_MOESM3_ESM.pdf]

Description of Additional Supplementary Files

File Name: **Supplementary Data 1**
Description: Datasets of this study
