# Supplementary material for: Fungal mycelium classified in different material families based on glycerol treatment
Source: Commun Biol. 2020 Jun 26;3:334. doi: 10.1038/s42003-020-1064-4 (PMC7320155; doi:10.1038/s42003-020-1064-4)
Supplement: Supplementary file 5 — Reporting Summary [file 42003_2020_1064_MOESM5_ESM.pdf]

nature research

Corresponding author(s): Han Wosten

Last updated by author(s): Jan 15, 2020

Reporting Summary

Nature Research wishes to improve the reproducibility of the work that we publish. This form provides structure for consistency and transparency in reporting. For further information on Nature Research policies, see Authors & Referees and the Editorial Policy Checklist.

Statistics

For all statistical analyses, confirm that the following items are present in the figure legend, table legend, main text, or Methods section.

n/a	Confirmed	
☐	☒	The exact sample size (n) for each experimental group/condition, given as a discrete number and unit of measurement
☐	☒	A statement on whether measurements were taken from distinct samples or whether the same sample was measured repeatedly
☐	☒	The statistical test(s) used AND whether they are one- or two-sided<br>*Only common tests should be described solely by name; describe more complex techniques in the Methods section.*
☒	☐	A description of all covariates tested
☐	☒	A description of any assumptions or corrections, such as tests of normality and adjustment for multiple comparisons
☐	☒	A full description of the statistical parameters including central tendency (e.g. means) or other basic estimates (e.g. regression coefficient) AND variation (e.g. standard deviation) or associated estimates of uncertainty (e.g. confidence intervals)
☐	☒	For null hypothesis testing, the test statistic (e.g. F, t, r) with confidence intervals, effect sizes, degrees of freedom and P value noted<br>*Give P values as exact values whenever suitable.*
☒	☐	For Bayesian analysis, information on the choice of priors and Markov chain Monte Carlo settings
☒	☐	For hierarchical and complex designs, identification of the appropriate level for tests and full reporting of outcomes
☒	☐	Estimates of effect sizes (e.g. Cohen's d, Pearson's r), indicating how they were calculated

Our web collection on statistics for biologists contains articles on many of the points above.

Software and code

Policy information about availability of computer code

Data collection	No software was used to collect data
Data analysis	Statistical analyses was performed with the software package IBM SPSS statistics 22.0 (IBM Corporation, Armonk, New York)

For manuscripts utilizing custom algorithms or software that are central to the research but not yet described in published literature, software must be made available to editors/reviewers. We strongly encourage code deposition in a community repository (e.g. GitHub). See the Nature Research guidelines for submitting code & software for further information.

Data

Policy information about availability of data

All manuscripts must include a data availability statement. This statement should provide the following information, where applicable:

- Accession codes, unique identifiers, or web links for publicly available datasets
- A list of figures that have associated raw data
- A description of any restrictions on data availability

Figures 2 and 3, Table 1, Supplementary Table 1 and Supplementary Figure 1 have associated raw data that are listed in the Supplementary Data file that we have submitted

Field-specific reporting

Please select the one below that is the best fit for your research. If you are not sure, read the appropriate sections before making your selection.

☒ Life sciences ☐ Behavioural & social sciences ☐ Ecological, evolutionary & environmental sciences

Life sciences study design

All studies must disclose on these points even when the disclosure is negative.

Sample size	Sample size was not calculated via statistical design. The number of samples was based on what was reasonable regarding the outline of this report.
Data exclusions	One specimen was excluded as it failed prior the mechanical analyses. The thickness and weight was determined for this specimen but these data were also excluded from the datafile.
Replication	Experiments for thickness, weight and mechanical analyses were repeated at least two times and with reproducable results. The water contact angle was measured by means of 15 measurements for each of the four biological replicates. From this, the means were taken to calculate the average water contact angle.
Randomization	Care was taken that all specimens within the experimental groups of the glycerol treatment experiment originated from seperate cultures grown in seperate erlenmeyers in 3 different occasions.
Blinding	The experiments performed in our study did not ask for blinding because of the absence of a preferred outcome.

Reporting for specific materials, systems and methods

We require information from authors about some types of materials, experimental systems and methods used in many studies. Here, indicate whether each material, system or method listed is relevant to your study. If you are not sure if a list item applies to your research, read the appropriate section before selecting a response.

Materials & experimental systems

n/a	Involved in the study	
☒	☐	Antibodies
☒	☐	Eukaryotic cell lines
☒	☐	Palaeontology
☒	☐	Animals and other organisms
☒	☐	Human research participants
☒	☐	Clinical data

Methods

n/a	Involved in the study	
☒	☐	ChIP-seq
☒	☐	Flow cytometry
☒	☐	MRI-based neuroimaging
